# Vitamin D content and prevalence of vitamin D deficiency in patients with epilepsy: a systematic review and meta-analysis

**DOI:** 10.3389/fnut.2024.1439279

**Published:** 2024-08-30

**Authors:** Yuanyuan Liu, Chao Gong, Jiawei Li, Xin Ning, Pei Zeng, Luchuan Wang, Beibei Lian, Jiahao Liu, Liya Fang, Jin Guo

**Affiliations:** ^1^College of Rehabilitation Medicine, Jiamusi University, Jiamusi, China; ^2^Jiamusi University Affiliated No. 3 Hospital, Jiamusi, China; ^3^Jiamusi University Affiliated No. 1 Hospital, Jiamusi, China

**Keywords:** vitamin D, epilepsy, vitamin D deficiency, seizure, anti-seizure medications

## Abstract

**Introduction:**

The prevalence of vitamin D deficiency and vitamin D levels in patients with epilepsy (PWE) were systematically evaluated, and the differences between subgroups were analyzed.

**Method:**

We identified all articles investigating the prevalence of vitamin D deficiency in patients with epilepsy from the database established in March 2024 from PubMed, Web of Science, and Embase. We divided them into anti-seizure medication (ASM) interventions and non-ASM interventions according to whether or not someone used ASM.

**Results:**

A total of 68 articles were included. The prevalence of newly diagnosed epilepsy was 50.2% (95% CI: 38.7–61.7%), and the prevalence after ASM intervention was 47.9% (95% CI: 40–55.9%), including 7,070 patients with epilepsy. Subgroup and meta-regression analyses were performed according to the diagnostic criteria, economic development level, region, age, ASM treatment, and other factors. The results showed that the differences were not significant. In addition, the vitamin D content of epilepsy patients (18.719 ng/mL) was lower than that of healthy people (20.295 ng/mL).

**Conclusion:**

The prevalence of vitamin D deficiency in patients with epilepsy is very high. Still, the related factors have little effect on the high prevalence of vitamin D in epilepsy, and ASM intervention can reduce the vitamin D content in patients with epilepsy. Therefore, it is emphasized that monitoring vitamin D levels is part of the routine management of patients with epilepsy.

**Systematic review registration:**

The protocol was registered with the International Prospective Register of Systematic Reviews (PROSPERO). (registration number CRD42023493896). https://www.crd.york.ac.uk/PROSPERO/ # myprospero.

## 1 Introduction

Vitamin D (VitD) is a fat-soluble vitamin that exists in many forms, mainly in the form of ergocalciferol (VitD_2_) and cholecalciferol (VitD_3_) (1, 2). VitD is essential throughout life. It participates in bone formation and plays a vital role in several other physiological systems. Adding VitD can prevent a variety of degenerative diseases (2). In addition, several studies have shown that VitD deficiency is associated with behavioral problems such as schizophrenia, multiple sclerosis, and epilepsy (3).

Epilepsy is one of the most common neurological diseases, regardless of age, race, social class, geography, or national boundaries (4). The current prevalence of epilepsy continues to rise, with about 2.4 million people newly diagnosed each year (5). It is estimated that nearly 6 out of every 1,000 children in the world have epilepsy, and the incidence of epilepsy in children is about twice that of adults (6). In the past decade, the prevalence of Vit D deficiency has been increasing worldwide, but this problem has been neglected (7). Vit D deficiency is a major global public health problem. Usually, 25-(OH)D vitamin levels are used as reference data to determine the status of Vit D (8). Globally, the prevalence of Vit D deficiency (25-(OH)D < 20 ng/mL) is estimated to be 24–40% in the United States and Europe (9). In Malaysia, the total proportion of Vit D deficiency is about 64% (10). The relationship between Vit D deficiency and epilepsy has been studied. The prevalence of epilepsy in adults with Vit D deficiency is 31 to 45% (11, 12). Vit D deficiency is not only common in children with epilepsy (22-25%) (13–15)but also common in the elderly (16).

## 2 Method

This meta-analysis was performed following the guidelines outlined in the Preferred Reporting Items for Systematic Reviews and Meta-analysis (PRISMA) checklist (17). The protocol was registered with the International Prospective Register of Systematic Reviews (PROSPERO) (registration number CRD42023493896).

### 2.1 Search strategy

We systematically searched three databases (PubMed, Embase, and Web of Science) to determine the prevalence of Vit D deficiency in patients with epilepsy from the establishment of the database to March 2024.

We used the following search terms: epilepsy, epilepsies, seizure disorder, seizure disorders, seizures, vitamin D (Vit D, Vitamin D, V D), prevalence, epidemic, epidemiology, and incidence. The search was performed by combining MeSH words, titles/abstracts and combined according to Boolean logic principles (using AND, OR, or NOT). We use Endnote 20 to manage and delete duplicate articles. The reference lists of selected articles were also manually retrieved. Take Pubmed as an example; see Table 1. See Supplementary material S1 for search terms.

**Table 1:** The specific retrieval method of Pubmed.

Steps	Search strategy
#1	“Epilepsy”[Mesh]
#2	(((Epilepsies[Title/Abstract]) OR (Seizure Disorder[Title/Abstract])) OR (Seizure Disorders[Title/Abstract])) OR (Seizures[Title/Abstract])
#3	(“Epilepsy”[Mesh]) OR ((((Epilepsies[Title/Abstract]) OR (Seizure Disorder[Title/Abstract])) OR (Seizure Disorders[Title/Abstract])) OR (Seizures[Title/Abstract]))
#4	“Vitamin D”[Mesh]
#5	(Vit D) OR (VD)
#6	(“Vitamin D”[Mesh]) OR ((Vit D) OR (VD))
#7	#3 AND #6

### 2.2 Inclusion and exclusion criteria

#### 2.2.1 Inclusion criteria

1. Articles reporting the prevalence of epilepsy with VitD deficiency, directly or indirectly.
2. English articles and Chinese articles.
3. Articles containing original data: a cross-sectional study, a case–control study, and a cohort study.

#### 2.2.2 Exclusion criteria

1. Reviews, systematic reviews, meta-analyses, meeting summaries, student papers, comments, or letters.
2. The full text could not be obtained.
3. Article data were missing, repeated, or unavailable.
4. Conference abstracts or unpublished articles.

### 2.3 Article selection and data extraction

Two reviewers screened the retrieved articles by reading titles and abstracts according to the above criteria to exclude unrelated and repetitive articles, then read the full texts of the remaining publications independently. Finally, they discussed differences with a third expert to reach a consensus. We extracted the following data separately from each article: first author, publication year, case collection time, study type, total number of epilepsy patients, number of patients with Vit D deficiency, prevalence rate, age, judgment standard, economic status and region, publication time, Vit D content, ASM treatment (monotherapy or multidrug therapy), diagnostic criteria for epilepsy developed by the International League Against Epilepsy (ILAE) and the proportion of women. If necessary, the study authors were contacted for more data or clarification. After discussion, we resolved the disagreement by consensus with the third senior reviewer.

### 2.4 Quality assessment

We used the tools of Hoy et al. (18) to assess the risk of bias in studies measuring the prevalence of Vit D deficiency in patients with epilepsy. The tool included 10 items to judge the risk of bias: (1) selection-related bias, (2) bias associated with non-response, (3) measurement-related bias, and (4) bias associated with analysis. Each item included two options: (1) high or low risk. The high-risk option was chosen if the study lacked basic information to make the judgment. The overall assessment of the risk of bias was rated as low (three or fewer high-risk items), moderate (four to five high-risk items), and high (six or more high-risk items) risk bias. Two reviewers individually reviewed each study. The disagreement was resolved by consensus and in consultation with the senior reviewer. The detailed results of the quality assessment are shown in Supplementary material S2.

### 2.5 Data synthesis and analysis

Stata 17.0 (Stata Corp., College Station, TX, United States) was used to analyze the prevalence of Vit D deficiency in people with epilepsy and provide its 95% confidence interval (CI). The Q test analyzed the heterogeneity among the included studies, and *I*^2^ quantitatively judged the heterogeneity. If the heterogeneity was high (*I*^2^ ≥ 50%), the source of heterogeneity was further analyzed. After excluding the influence of articles with obvious heterogeneity, the random effect model (REM) was used for meta-analysis. Otherwise, the fixed effect model (FEM) was used. In addition, subgroup analysis and meta-regression analysis were performed according to relevant factors to explore the sources of heterogeneity and the differences in the prevalence of epilepsy among subgroups. Related factors include diagnostic criteria, economic development level, region, age, and ASM treatment (single drug treatment; multidrug therapy).

## 3 Results

### 3.1 Article selection

**Figure 1:**
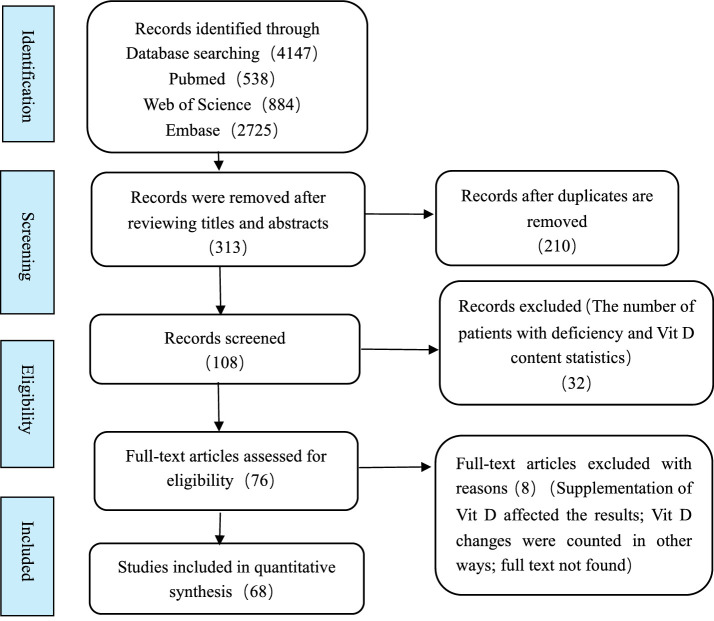
Flowchart of the study selection.

Four thousand one hundred forty-seven were retrieved: PubMed: 538, Embase: 2725, Web of Science: 884. After eliminating duplicate articles, the titles and abstracts of each article were screened. After reading the full text, a total of 76 articles were evaluated as eligible for inclusion using the criteria specified. Among them, 68 studies were included in the meta-analysis after a detailed evaluation. The research selection process is shown in Figure 1.

### 3.2 Article characteristics

The essential characteristics of the included literature are shown in Table 2. The 68 articles included in this meta-analysis were published from 2014 to 2024, involving 25 countries and regions worldwide, and the data collection time was from 1994 to 2020. The number of epilepsy patients in the article ranged from 16 to 524, for a total of 3,016 people. The quality scores of the articles ranged from 1 to 5 points, of which 4 were low, and 64 were moderate. A detailed description of the article is shown in Table 2. Detailed quality assessment results are shown in Supplementary material S3.

**Table 2:** Basic characteristics of literature.

First author	Published Time	Countries or regions	Time range of data collection	Epilepsy	Vit D deficient < 20 ng/mL	Vit D deficient < 15 ng/mL	Prevalence (%) deficient	Age (years)M (SD)	Proportion of female (N)	Diagnostic criteria of epilepsy	Quality score
Nirmal	2024	India	2020.8–2020.9	30	–		–	4 ~ 10 years old	60%(21)	–	3
Prusty	2024	India	–	37	18		48.65	newborn	45.7(17)	–	3
Leandro-Merhi	2023	America	2020.10–2021.12	93	31		33	46.5 ± 15.1 years old	61.3(57)	ILAE in 2017	1
Khoo	2023	Malaysia	2021.12–2022.8	159	73		45.9	≥18 years old	46.54(74)	ILAE	3
Mishra	2023	India	2019.2–2020.4	16	2		12.5	2–12;.7.1 ± 2.8 years old	50(8)	ILAE in 2017	3
Moraes	2023	Brazil	2020	32	13		40.6	≥18 years old	53.1(17)		3
Al haidari	2022	Saudi Arabia	2016.11–2020.4	524	455		86.8	>14 years old	42.7(224)	ILAE in 2017	3
Winterhalder	2022	England	2015–2017	102	76		74.5	39 ± 15.3 years old	39.4(41)	–	3
Likasitthananon	2022	Thailand	2018.4–2019.1	138	98		71.01	1 ~ 20 years old	46.4(64)	–	3
Reddy	2021	India	2018.11–2020.08	30		16	53.33	35–40 weeks old.	–	–	3
Cunha	2021	Portuguese	2017.9–2018.4	92	52		56.5	≥18 years old	55.4(51)	ILAE in 2017	3
Kong	2020	Malaysia	2014.4–2015.4	239		55	23	3.7–18.8 years old	40.6(97)	–	3
Specht	2020	Australia	1994–2002	403	–		–	1–4 years old	46.9(189)	–	2
Jésus	2020	France	–	46	40		87	15.3 ± 9.9 years old	75.22(35)	–	2
Elmazny	2020	Egypt	2018.5–2018.8	42	17		40.47	8–18 years old	54.8(23)	ILAE in 2017	3
Bhat	2020	India	2015.11–2018.10	223	69		30.9	7–59 months old	46.64(104)	–	3
Papassava	2019	Greece	12 months	54	21		38	9 ± 4.5 years old	44.4(24)	–	3
Viraraghavan	2019	India	2011.11–2013.3	29	13		46	5–10 years old	20.69(6)	–	2
Suljic	2018	Bosnia and Herzegovina	12 months	50	–		–	36.7 ± 10.25 years old	60(30)	–	3
Tombini	2018	Italy	2014–2016	160	120		75	50.6 ± 19.3 years old	61.25(98)	–	3
Fong	2018	Malaysia	2014.4–2015.4	244		55	22.5	3.7–18.8 years old	40.16(98)	–	3
Albaghdadi	2016	Syria	2015.4–2016.4	50	49		98	26 ± 7.2 years old	66(33)	–	3
Nagarjunakonda	2016	India	2013.7	98	38		41	≥18 years old	50(49)	–	3
Paticheep	2015	Thailand	2012.10–2013.9	30	7		23.3	3–18 years old	56.67(17)	–	3
Lee	2015	Thailand	12 months	143	81		56.6	7.4 ± 5.4 years old	28(40)	–	3
Teagarden	2014	America	2008.1–2011.7	596	268		45	18–81 years old	56(334)	–	3
Ramelli	2014	Switzerland	2013.1–2013.5	58	32		55	2–20 years old	41.38(24)	–	3
Baek	2014	Korea	2012.6–2012.11	143	13		9.1	2–17 years old	37.06(53)	–	3
Fong	2014	Australia	2010.1–2011.8	111	24		22	3–18 years old	57.7(64)	–	3
Razazizan	2013	Iran	6 months	48	20		41.6	2–14 years old	47.92(46)	–	3
Cesur	2013	Turkey	–	91	37		40.7	6.01 ± 4.06 months old	23.1(21)	–	3
Misra	2010	India	6 months	32	11		34	2–12 years old	43.75(14)	–	3
Mehrotra	2010	India	2006.4–2007.3	60	54		96.9	0.5–6 months old	46.67(32)	–	3
Verrotti	2010	Italy	12 months	20	–		–	16–22 years old	0	–	3
Phabphal	2009	Thailand	2004.4–2006.12	123	4		3.3	20–50 years old	50.4(62)	–	3
Nettekoven	2008	Germany	2005.12–2006.7	38	29		79.3	5–12 years old	34.3(13)	–	3
Cansu	2008	Turkey	18 months	34	–		–	1–18 years old	–	ILAE	3
Bergqvist	2007	America	2015	45	2		4	5.1 ± 2.7 years old	27(12)	–	3
Kulak	2004	America	2001.5–2003.1	58	20		34.4	34.4 ± 6 years old	69(40)	–	3
Farhat	2002	America	1998.11–2020.1	71	50		70.42	5–64 years old	47.89(34)	–	3
Gunawan	2023	Indonesia	6 months	60	30		50	7.6 ± 3.42 years old	50(30)	–	3
Khalifah	2022	Saudi Arabia	2017.12–2021.3	159	72		45.28	9 ± 3.5 years old	46.54(74)	–	3
Mohammadi	2022	Iran	2012.2–2012.8	45	21		46.75	17.9 ± 9.97 months old	51.1(23)	–	3
Dong	2022	China	2019.1–2021.6	648	547		49.19	3–8 years old	45.06(292)	–	3
Saket	2021	Iran	2016–2017	60	6		10	5.72 ± 2.93 years old	53.33(32)	–	3
Papassava	2021	Greece	12 months	42	18		43.2	11.9 ± 4.6 years old	–	–	3
Fathima	2021	India	2018.12–2019.12	50	5		6.7	child	–	–	3
Qiu	2021	China	2017.11–2018.6	25	8		32	0–3 years old	52(13)	–	3
Heydarian	2020	Iran	2016.12–2018.4	53	–		–	6–60 months old	49.1(26)	–	3
Azad	2020	India	2015.8–2016.7	200	106		53	1–18 years old	40(80)	ILAE in 2017	3
Kamili	2020	India	2018.9–2019.2	30	23		73	1–14 years old	26.67(8)	–	3
Abdullah	2020	Iraq	2019.3–2019.9	50		49	98	7.57 ± 3.62 years old	56(28)	–	3
Durá-Travé	2018	Spain	2013.10–2014.6	90	25		27.78	1–18 years old	56.67(51)	ILAE	3
Sreedharan	2018	India	2012.6–2013.5	56		9	16.07	2–13 years old	50(28)	–	3
Shellhaas	2010	America	2008.9–2009.3	78	20		25	11.64 ± 4.37 years old	59(46)	–	3
Kaul K	2023	England	2022.9–2023.10	43	17		62.8	33.2 ± 16.7 years old	48.5(66)	–	3
Kiran	2023	India	24 months	150	110		73.3	0.5–5 years old	18(27)	–	3
Arimbawa	2023	Bali Island	2016.5–2016.9	30	–		–	1–11 years old	36.67(11)	–	3
Magsi	2022	Pakistan	2021.7–2022.1	50	28		56	18–30 years old	48(24)	–	3
Kamil	2019	Iraq	2017.2–2018.2	40	15		37.5	18–45 years old	55(22)	ILAE	3
Singla	2017	India	–	25	21		84	37.7 ± 13.9 years old	40(10)	–	3
Lee	2015	Korea	–	198	124		62.6	11.5 ± 4.4 years old	48.5(96)	–	3
Yaghini	2015	Iran	2011.1–2011.3	30		14	47	7.9 ± 2.4 years old	–	–	3
Patil	2015	India	2012.8–2014.7	70	50		71.4	2–16 years old	40(28)	–	3
Sonmez	2015	Turkey	2011.9–2012.11	60	48		80	5–16 years old	56.67(34)	–	3
Khoo	2015	Korea	2009.1–2010.4	41	–		–	28.2 ± 8.4 years old	46.3(19)	–	3
Koshy	2014	India	–	55	32		58.1	27.7 ± 7.9 years old	0	–	3
Zelleke	2012	Turkey	2013.3–2013.6	40	25		62.5	6.65 ± 5.29 years old	57.5(23)	–	3

### 3.3 Prevalence of Vit D deficiency

**Figure 2:**
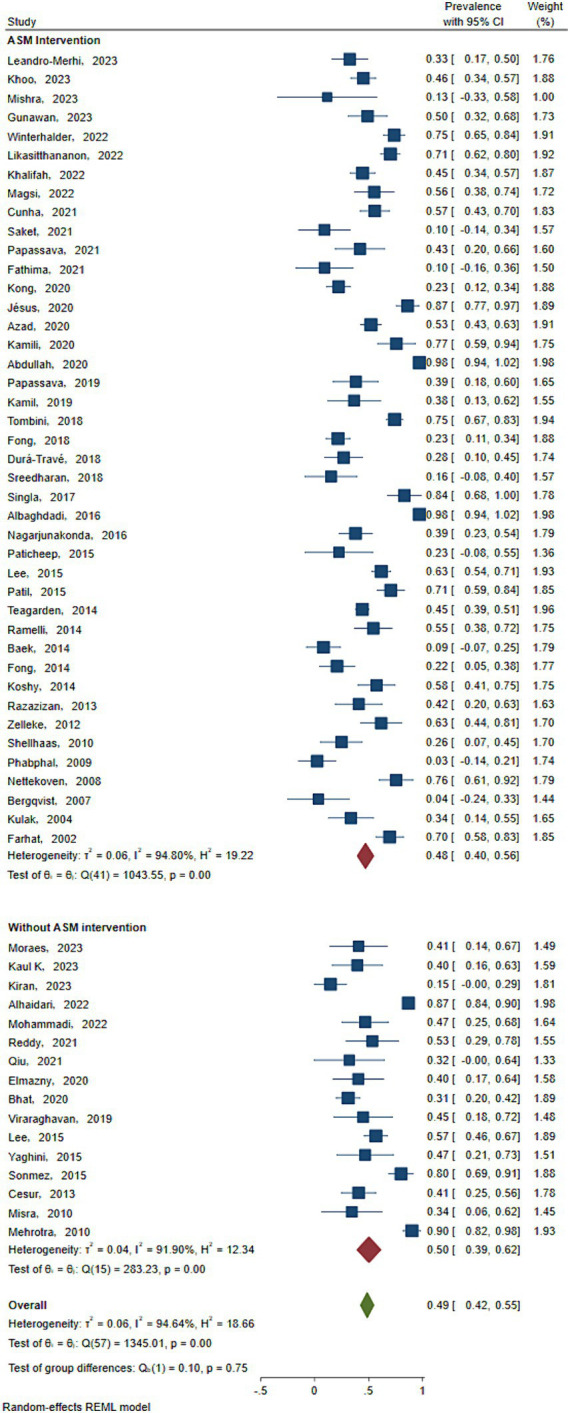
Prevalence rate. Horizontal line: indicates the confidence interval for this study. Square: indicates the effect size of the study; and the size of the block represents the weight, that is, the contribution to the meta-analysis. Green diamond: represents the combined effect size. Test of group difference *p*: *p*-value representing the combined effect size.

As shown in Figure 2, the prevalence of Vit D deficiency in epilepsy was estimated according to the literature. A total of 2,167 patients were included in this study. The number of people with VitD deficiency at the new diagnosis of epilepsy was 698, the total prevalence of epilepsy in Vit D deficiency was 50.2% (95% CI: 38.7–61.7%). The number of people who developed VitD deficiency after ASM intervention was 208, and the prevalence after ASM intervention was 47.9% (95% CI: 40 -55.9%). Significant heterogeneity was found in the study (*I*^2^ = 89.95%, *p* < 0.001).

### 3.4 Vit D content analysis

**Figure 3:**
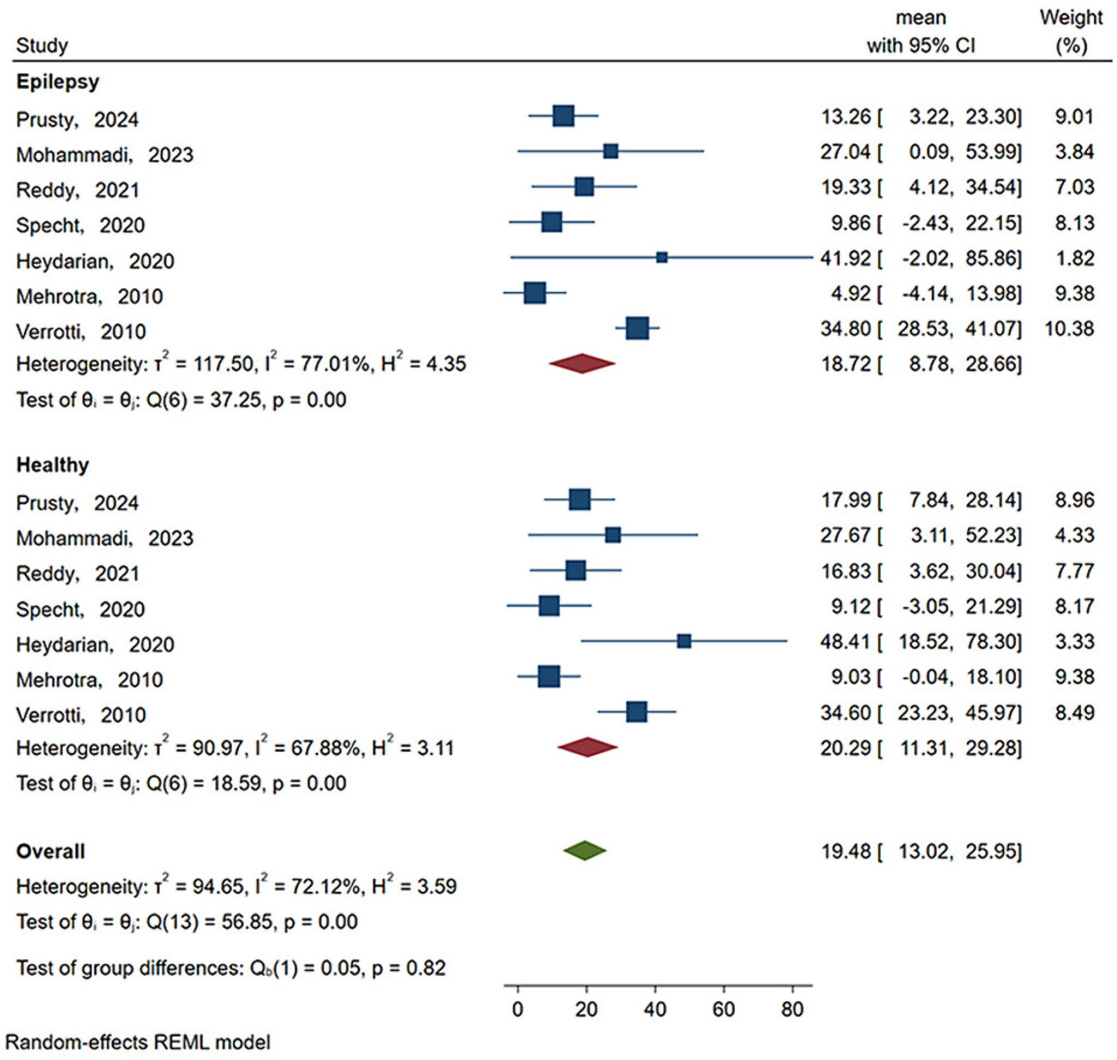
Vit D content in epilepsy patients and healthy people. Horizontal line: indicates the confidence interval for this study. Square: indicates the effect size of the study; and the size of the block represents the weight, that is, the contribution to the meta-analysis. Green diamond: represents the combined effect size. Test of group difference *p*: p-value representing the combined effect size.

**Figure 4:**
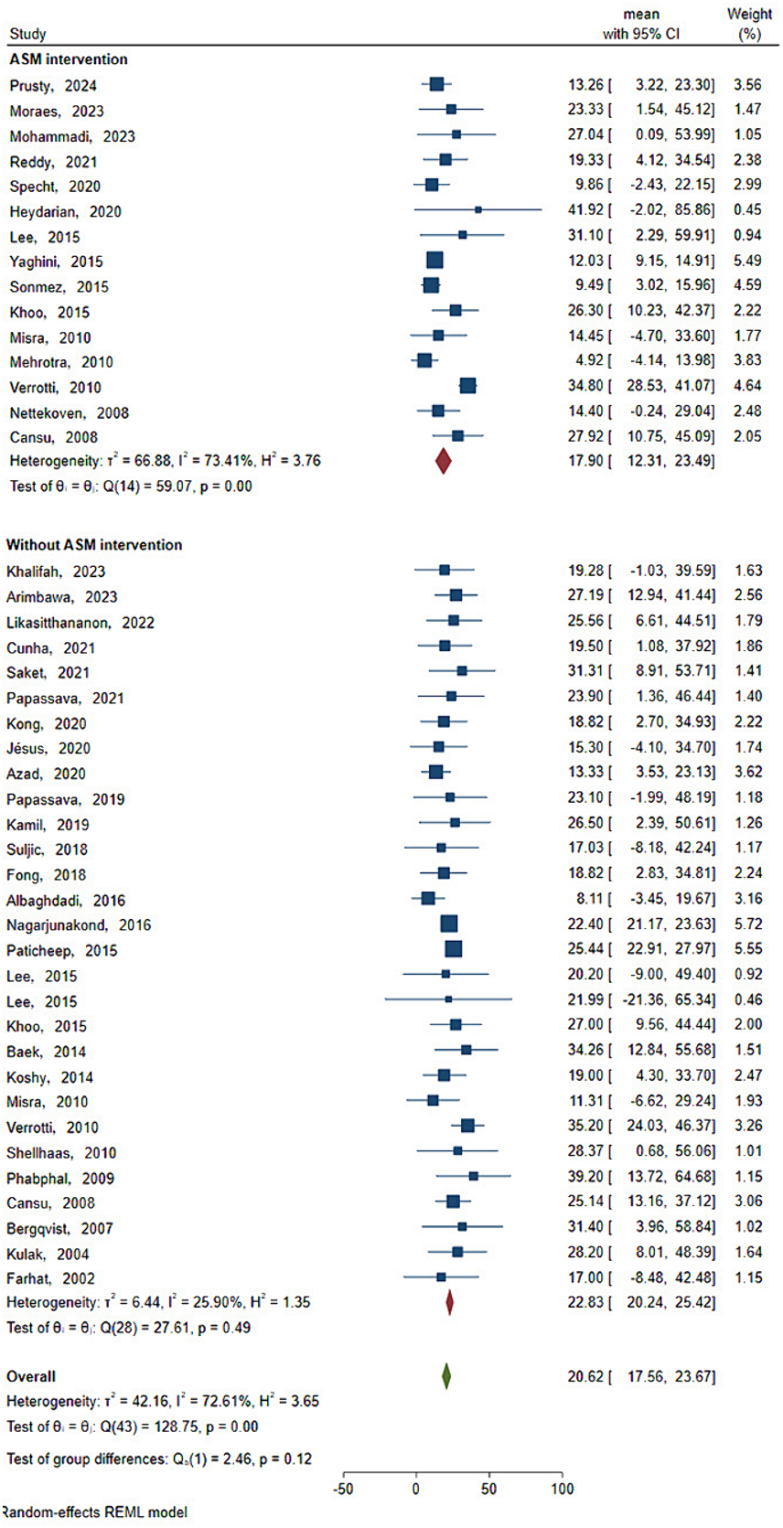
Vit D content before and after ASM intervention. Horizontal line: indicates the confidence interval for this study. Square: indicates the effect size of the study; and the size of the block represents the weight, that is, the contribution to the meta-analysis. Green diamond: represents the combined effect size. Test of group difference *p*: p-value representing the combined effect size.

As shown in Figure 3 and Table 3, the statistical results showed that the Vit D content (18.719 ng/mL) of epilepsy patients without ASM intervention was lower than that of healthy people (20.295 ng/mL). As shown in Figure 4 and Table 3, the Vit D content (17.9 ng/mL) of epileptic patients after ASM intervention was lower than that before ASM intervention (22.828 ng/mL).

**Table 3:** Vit D content analysis.

Subgroup	Number of studies	*N*	Event	*I*^2^(%)	*p*-value	(95% CI)		Mean(ng/mL)
VitD content								
Healthy	7	648	109	67.88	0	11.31	29.279	20.295
Epilepsy	7	648	109	77.01	0	8.778	28.661	18.719
Without ASM intervention	15	1,538	698	25.9	0.49	20.238	25.418	22.828
ASM intervention	29	629	208	73.41	0	12.31	23.494	17.902

### 3.5 Subgroup analysis of the prevalence of Vit D deficiency in patients with epilepsy

As shown in Table 4, based on the newly diagnosed epilepsy patients without ASM intervention, in terms of diagnostic criteria, Vit D deficiency was defined as less than 15 ng/mL, the prevalence of Vit D deficiency in patients with epilepsy (33.5, 95% CI: 19.9–47.1%) was lower than the criterion of less than 20 ng/mL, the prevalence of Vit D deficiency in patients with epilepsy (56.5, 95% CI: 44.1–69%) (*p* < 0.05).In terms of age, the prevalence of Vit D deficiency in epilepsy patients aged ≥18 years (57.8, 95% CI: 25.3–90.3%) was the highest, followed by the prevalence of Vit D deficiency in epilepsy patients aged 5–18 years (52.3, 95% CI: 29.6–74.9%), and the prevalence of Vit D deficiency in epilepsy patients aged 0–5 years was the lowest (46.7, 95% CI: 32.9–60.6%). In terms of economic status and region, the prevalence of vitamin D deficiency in epileptic patients was highest in Europe (54.5, 95% CI: 27.4–81.6%), followed by the Asian group (49.5, 95% CI: 35.6–63.5%) and lowest in the American group (40.6, 95% CI: 13.9–67.3%).

**Table 4:** Subgroup analysis and meta-regression analysis of the prevalence of epilepsy comorbid Vit D deficiency without ASM intervention.

Subgroup	Number of study	*N*	Event	*I*^2^(%)	*p*-value	(95% CI)		Prevalence(%)	Results of meta-regression for prevalence of epilepsy		
									*p*-value	Exp (b)	(95% CI)
Criteria											
< 15 ng/mL	5	465	132	60.37	0.05	19.9	47.1	33.5	0.064	0.800431	0.6313566–1.014782
<20 ng/mL	12	1,131	782	91.2	0	44.1	69	56.5			
Age											
0 ~ 5	10	834	340	87.5	0	32.9	60.6	46.7	0.407	1.062882	0.9125689–1.237955
5 ~ 18	4	163	89	77.64	0	29.6	74.9	52.3			
≥18	3	599	485	89.8	0	25.3	90.3	57.8			
Economic status and region											
Americas	1	32	13	/	/	13.9	67.3	40.6	0.964	1.004924	0.800347–1.261751
Asia	12	1,333	791	93.19	0	35.6	63.5	49.5			
Europe	3	194	102	88.09	0	27.4	81.6	54.5			

As shown in Table 5, in patients with epilepsy based on ASM intervention,in terms of diagnostic criteria, Vit D deficiency was defined as less than 15 ng/mL, the prevalence of Vit D deficiency in patients with epilepsy (40.6, 95%CI: 1.8–79.4%) was lower than the criterion of less than 20 ng/mL, the prevalence of Vit D deficiency in patients with epilepsy (48.8, 95% CI: 40.9–56.7%). In terms of age, the prevalence of Vit D deficiency in epilepsy patients aged ≥18 years (55, 95% CI: 41.5–68.6%) was the highest, followed by the prevalence of Vit D deficiency in epilepsy patients aged 5–18 years (45.4, 95% CI: 34.9–55.9%), and the prevalence of Vit D deficiency in epilepsy patients aged 0–5 years was the lowest (37.7, 95% CI: 9.8–65.5%). In terms of economic status and region, the prevalence of vitamin D deficiency in epileptic patients was highest in Europe (61, 95% CI: 49.3–72.8%), followed by the Asian group (46.3, 95% CI: 35.1–57.5%) and lowest in the American group (37.7, 95% CI: 20.9 -54.5%). The prevalence of Vit D deficiency in patients with epilepsy treated with monotherapy (46.5, 95 %CI: 26.2–66.9%) was lower than that in patients with epilepsy treated with multi-drug therapy (51.6, 95% CI: 37.6–65.5%).

**Table 5:** Subgroup analysis and meta-regression analysis of the prevalence of epilepsy comorbid with Vit D deficiency treated with ASM.

Subgroup	Number of study	*N*	Event	*I*^2^(%)	*p*-value	(95% CI)		Prevalence(%)	Results of meta-regression for prevalence of epilepsy		
									*p*-value	Exp (b)	(95% CI)
Criteria											
<15 ng/mL	4	589	168	98.34	0	18	79.4	40.6	0.605	0.931462	0.7071958–1.22678
<20 ng/mL	38	3,576	1,669	93.12	0	40.9	56.7	48.8			
Age											
0 ~ 5	7	321	119	94.02	0	9.8	65.5	37.7	0.195	1.084469	0.9577022–1.228014
5 ~ 18	20	2072	841	91.15	0	34.9	55.9	45.4			
≥18	15	1772	877	95	0	41.5	68.6	55			
Economic status and region											
Americas	6	941	391	87.30	0	20.9	54.5	37.7	0.886	1.008139	0.8997004–1.129647
Asia	25	2,391	984	96.02	0	35.1	57.5	46.3			
Europe	10	722	438	86.36	0	49.3	72.8	61			
ASM intervention method											
Monotherapy	6	467	200	89.81	0	26.2	66.9	46.5	0.723	1.046687	0.7916734–1.383846
Polytherapy	6	363	168	71.28	0	37.6	65.5	51.6			

### 3.6 Publication bias and sensitivity analysis

**Figure 5:**
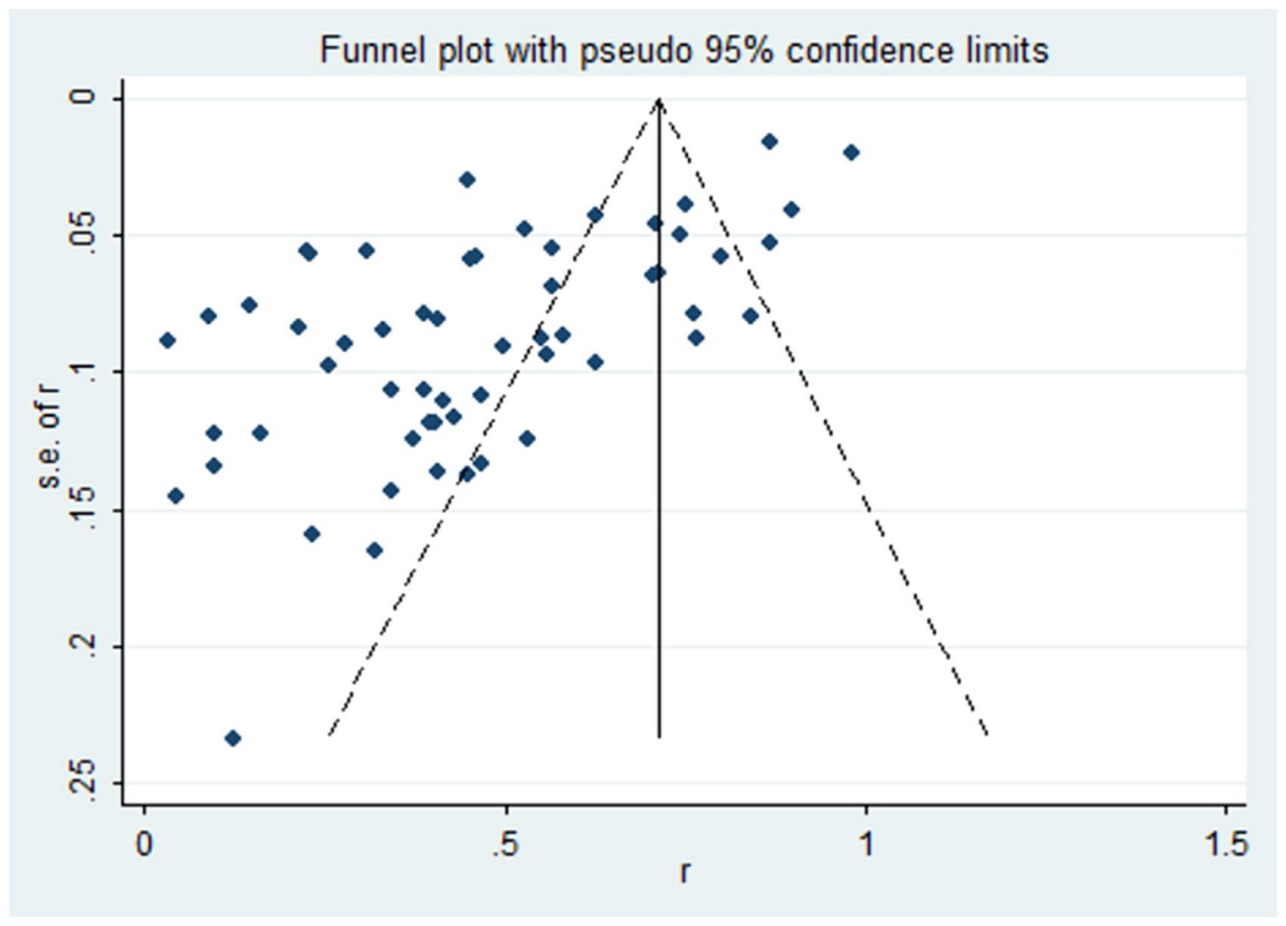
The funnel plot of the total prevalence of Vit D deficiency in epilepsy. Diamond square: represents the individual studies included. Horizontal axis: represents the effect size. Solid line in the middle: represents the merged OR value.

**Figure 6:**
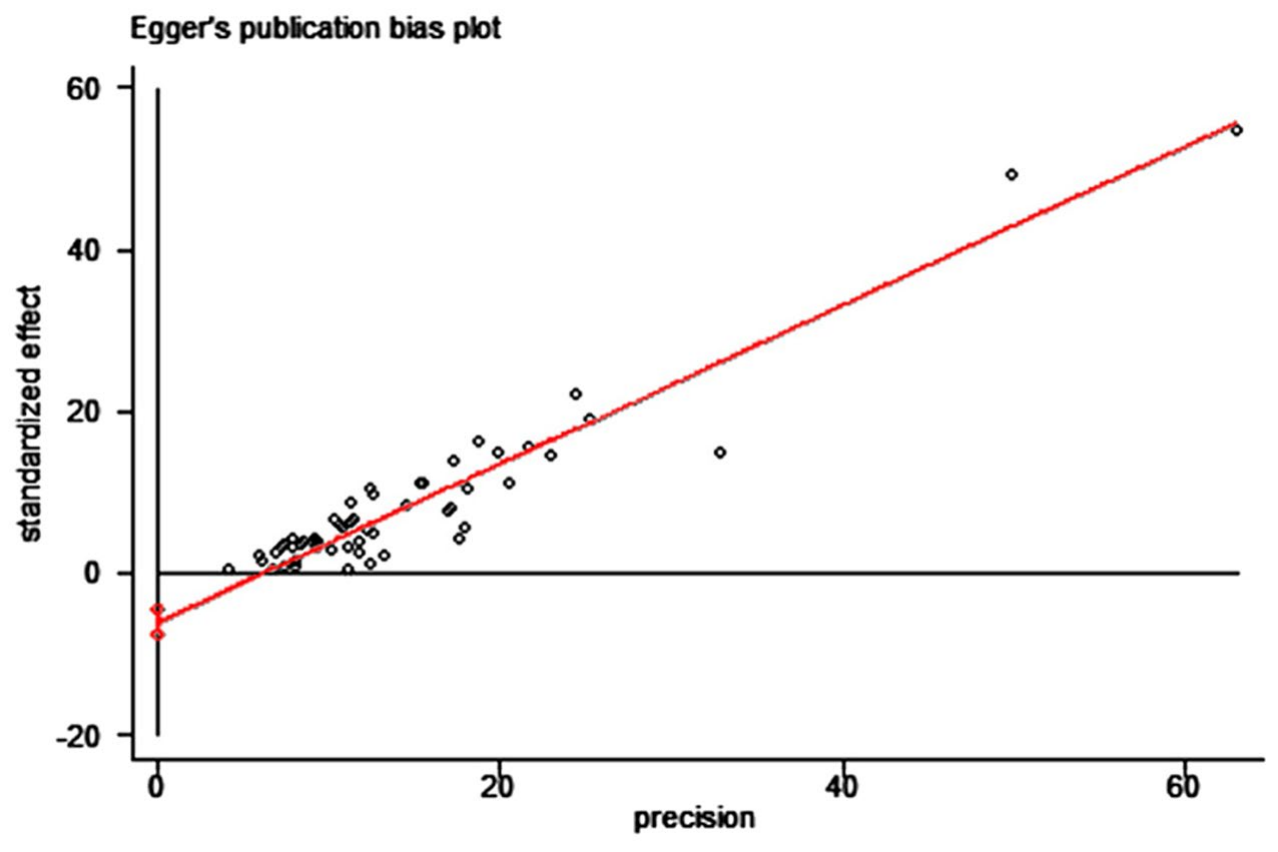
Egger’s test of the prevalence of Vit D deficiency in epilepsy.

We used the funnel plot to assess publication bias, as shown in Figure 5, revealing a significant asymmetry in the overall prevalence of Vit D deficiency. As shown in Figure 6, the Egger test showed a significant publication bias in the meta-analysis (*t* = −8.08, *p* < 0.01).

We performed sensitivity analysis using a single-article elimination method, and no articles were found to affect the overall results significantly. Meta-regression analysis was performed on the included literature, with the prevalence of Vit D deficiency in epilepsy as the dependent variable. The independent variables of the meta-regression analysis included the diagnostic criteria, age, economic development level and region, and ASM treatment (single-drug treatment, multidrug treatment).

## 4 Discussion

In this systematic review, 68 studies were included. We discussed the Vit D content of epileptic patients without ASM intervention, the Vit D content of healthy people, the Vit D content of epileptic patients after ASM intervention, and the prevalence of Vit D deficiency in epileptic patients before and after ASM intervention. The correlation between antiepileptic drugs, Vit D deficiency, and bone health in patients with epilepsy has been confirmed in previous studies (19). To understand whether epilepsy itself is related to the level of Vit D, it is necessary to study patients with untreated epilepsy. So far, the studies have been cross-sectional or longitudinal studies of patients who have been treated with antiepileptic drugs (20). Moreover, we sought to evaluate the longitudinal effects of antiepileptic drugs on serum 25-hydroxyVitD [25 (OH) D] levels and bone mineral metabolism parameters before and after ASM treatment. In addition, there are few studies on bone health in patients with epilepsy before the start of drug treatment (21–23). According to the literature, only a few studies have measured serum Vit D levels in untreated epileptic patients (24).

In the circulatory system, Vit D exists in the form of serum 25-(OH)D (25). Because it is a stable metabolite of Vit D (11), 25-(OH)D is the best marker for determining the state of Vit D in the human body. Complex methods for the measurement of 25-(OH)D include enzyme-linked immunosorbent assay (26), chemiluminescence assay (26, 27), GC–MS (28), HPLC-UV assay (29), radioimmunoassay (30, 31), and LC–MS / MS. (32–35) LC–MS / MS is the gold standard for determining 25-(OH)D. However it needs expensive high-end instruments and skilled technicians. Ion suppression from matrix effects, interference from C3 epimers, and forced derivatization to increase ionization are other well-known drawbacks that limit the use of this technique (36). In recent studies, using LC–MS/MS to quantify 25-(OH)D metabolites has good method performance characteristics and high sensitivity (37).

Our statistics show that the prevalence rate is 50.2%. The serum Vit D level of healthy children is relatively high, and the serum 25-(OH)D level is 20.295 ng/mL. However, the serum VitD level of epileptic children is relatively low, and the serum 25-(OH)D level is 18.719 ng/mL. The serum Vit D level in epileptic children is lower than that in healthy children, which may be related to epilepsy itself. There is evidence that seizures are the most common symptom in 78.7% of pediatric cases with 25-(OH)D deficiency (38). Therefore, there is a link between Vit D and epilepsy. Vit D also regulates specific brain genes—convulsive cytokines IL-6 and anti-convulsive growth factors, such as glial cell-derived neurotrophic factor and neurotrophin-3. In addition, Vit D plays an essential role in the regulation of calcium homeostasis and neuronal excitability. Increased cellular calcium promotes the release of excitatory neurotransmitters, leading to neuronal depolarization and the spread of seizures (39). Therefore, in the study of Chaudhuri et al. (40), the main pathogenesis of epilepsy seems to be based on the decrease of Vit D activity level, which may be caused by the ASM-induced cytochrome P450 enzyme, resulting in its conversion into inactive metabolites in liver microsomes. The anticonvulsant effect of Vit D was first reported by Christiansen et al. (38), and repeated in a small preliminary study in patients with drug-resistant epilepsy and low serum 25-(OH)D levels. In this study, supplementation with Vit D resulted in a 40% reduction in seizures. Studies of chemically induced seizures in Vit DR knockout mice have shown that there is a direct link between Vit DR gene ablation and increased susceptibility to seizures (41). However, there is also literature showing a lack of association between serum Vit D levels and any epileptic characteristics (42). There was no significant correlation between seizures (within 72 h and after 72 h) and VitD levels (*p* = 0.18) (43). After ASM intervention, the Vit D content of patients decreased. The statistical results of this paper showed that the Vit D content of patients without ASM (22.828 ng/mL) was higher than that of patients with ASM (17.902 ng/mL). Menon et al. (20) found that patients with standard 25-(OH)D levels, regardless of the use of any antiepileptic drugs, will enter various 25-(OH)D deficiency and insufficiency states within 6 months at the sub-therapeutic level of the drug. In a similar study, the average level of 25-(OH)D in the case group (28.79 ± 33.85) was lower than that in the control group (47.62 ± 46.16) (44). In the study of Viraraghavan et al. (45), the average serum 25-(OH)D level in patients with epilepsy was 22 ng/mL. Therefore, it is a fact that the serum Vit D level is significantly reduced in children with epilepsy.

However, some studies have found no relationship between 25-(OH)D deficiency and epilepsy (46–48). Therefore, we have a prevalence of 45%, and not all patients with epilepsy have Vit D deficiency at the beginning. However, whether the decrease of serum VitD level leads to epilepsy or epilepsy leads to the reduction of serum Vit D level, we cannot conclude the causal relationship between the two. It is worth noting that the role of Vit D in neuroprotection, brain development, and immune regulation has been confirmed in many studies (49), and because of the complex mode of action of VitD, its potential mechanism needs to be further studied to provide valuable insights into the pathophysiology of epilepsy.

However, the unreliability of commonly used Vit D measurements and the lack of consistency in the definition of ‘normal ‘populations have led to certain difficulties in establishing a reference range for serum 25-(OH)D concentrations (50), so commonly used Vit D measurements are not internationally standardized (30). There has yet to be a consensus on the definition of Vit D deficiency. The European Society of Pediatric Gastroenterology, Hepatology, and Nutrition recommended the use of 25-(OH)D serum concentration > 50 nmol/l (20 ng/mL) as sufficient and serum concentration < 25 nmol/l (10 ng/mL) as severe deficiency (51). Lawson Wilkins Pediatric Endocrine Society defined severe deficiency as <12.5 nmol/l (5 ng/mL), deficiency as <37.5 nmol/l (15 ng/mL), insufficiency as 37.5–50.0 nmol/l (15-20 ng/mL), and sufficiency as 50.0-250 nmol/l (20–100 ng/mL) (50). On the other hand, adult bone health studies have shown that the critical value is high, and the lower limit of 80 nmol/l (32 ng/mL) has been accepted as the lower limit of adult normal (52, 53). However, the American Medical Research Institute defined the 25- (OH) D threshold of 50 nmoL/L (20 ng/mL) as the normal value of the elderly (54).

Based on the newly diagnosed epilepsy without ASM intervention, this paper counted 17 articles, of which 5 articles defined Vi D deficiency as <15 ng/mL and 12 articles defined Vit D deficiency as <20 ng/mL. The prevalence of <15 ng/mL was 33.5%. The prevalence was 56.5% (*p* < 0.05) when <20 ng/mL was used as the standard. There was a significant difference in prevalence according to inconsistent cut-off values. When the definition of Vit D deficiency changed from <11 ng/mL to <20 ng/mL, the prevalence increased from 2 to 14% (55). Vit D deficiency was defined as less than 15 ng/mL, the prevalence of Vit D deficiency in patients with epilepsy is lower than the prevalence of Vit D deficiency in patients with epilepsy according to the standard of less than 20 ng/mL as a criterion for determining Vit D deficiency. The patients in the five articles were infants and children. If, according to the Lips criteria (56), all study and control infants had Vit D deficiency. However, even if the 25 (OH) D level < 10 ng/mL was used as the cut-off value, as reported in some early studies (57, 58), 90% of the study infants and 41.7% of the control infants would still be judged as Vit D deficiency (59). At the same time, Vit D levels are also affected by age, gender, geographical location, use of supplements, and BMI, further complicating the definition of Vit D deficiency and estimated prevalence (60).

Based on the epilepsy patients treated with ASM, we counted 42 articles in this paper. Among them, 4 articles defined Vit D deficiency as <15 ng/mL, and 38 articles defined Vit D deficiency as <20 ng/mL. The prevalence of <15 ng/mL was 40.6%. The prevalence of <20 ng/mL was 48.8%. Vit D deficiency was defined as less than 15 ng/mL, and the prevalence of Vit D deficiency in patients with epilepsy is lower than the prevalence of Vit D deficiency in patients with epilepsy according to the standard of less than 20 ng/mL as a criterion for determining Vit D deficiency. Moreover, the cut-off value of 25 (OH) D level was studied to distinguish seizure control. For example, the Leandro-Merhi et al. (61) study set the Vit D level to 20 mg / ml, and the chance of seizure control in patients using a single ASM was 6.99 times that of patients using two or more ASMs. The Vit D level was set to 40 mg/mL, and the number of ASM used did not interfere with the chance of control.

Malabanan et al. (62) proposed that the normal range of 25- (OH) D is greater than 20 ng/mL because levels below 20 ng/mL are usually associated with elevated PTH levels. Another study showed that a serum25- (OH) D level of 30 ng/mL is a more appropriate lower limit of average (63). It should be noted that patients may lack Vit D, and their 25- (OH) D levels are still within the laboratory reference range (64).

According to the statistical results of this paper, based on the newly diagnosed epilepsy without ASM intervention, in terms of age, the prevalence of Vit D deficiency in patients with epilepsy ≥18 years old was the highest, which was 57.8%. The prevalence of Vit D deficiency in patients with epilepsy aged 5–18 years old was 52.3%, and the prevalence of Vit D deficiency in patients with epilepsy aged 0–5 years old was the lowest, which was 46.7%. At the same time, after ASM intervention, in terms of age, the prevalence of Vit D deficiency in patients with epilepsy aged ≥18 years was the highest (55%), followed by the prevalence of VitD deficiency in patients with epilepsy aged 5–18 years (45.4%), and the prevalence of VitD deficiency in patients with epilepsy aged 0–5 years was the lowest (37.7%). Similar to previous reports, we hypothesize that puberty is a crucial factor for VitD deficiency. This is because puberty is a critical period of bone mineralization, which leads to high utilization of VitD and increased demand for VitD, resulting in a high deficiency rate (65). VitD levels in young people are usually low (66, 67). Young people typically work indoors in significant cities most of the day, while older people may be outdoors participating in physical exercise or leisure hobbies such as gardening (68). However, excessive exposure to sunlight does not further increase VitD production (50). Therefore, there are also reports that VitD levels in the elderly are low (69, 70). In theory, aging leads to lower levels of 7-dehydrocholesterol, which in turn reduces the synthesis of VitD3 (71). In addition, if bedridden, frail nursing home residents will not be able to get enough sunlight, reduce dietary intake, and rarely receive any vitamin supplements (72).

It has been reported that serum Vit D levels change significantly with age, Al Haidari et al. (73) found that there was a significant negative correlation between the age of onset and the level of Vit D (*r* = 0.26, *p* < 0.001,95% CI: 19% ~ 34%), and they found that there was a similar correlation between the age of seizures and the level of Vit D (*r* = 0.24, *p* < 0.001,95% Cl: 15% ~ 32%). In addition, in this experiment, when the age of treatment was divided into 20 years old as a group, the serum Vit D level of patients over 60 years old was significantly higher than that of patients under 20 years old (*p* < 0.01,95% CI: 17.7 ~ 37.7 nmol/L). A similar correlation was observed when comparing patients aged 41–60 years with patients under 20 years, but the degree was lower (high 9.3 nmol/L, *p* = 0.01,95% Cl: 2.1–16.6 nmol/L). Other patient characteristics did not show any correlation with serum Vit D levels (66). In this paper, the prevalence of 0 ~ 5-year-old group was 52%. Similar to the results of Nicolaidou et al. (74), the prevalence of Vit D deficiency in children with epilepsy was as high as 62.6%. Therefore, Vit D level is affected by age (75). Lee and Yu reported that Vit D deficiency is more common in epileptic adolescents >12 years of age. However, regardless of age, even if the Vit D level is normal at the beginning, the children with epilepsy will become insufficient with the extension of medication time during the follow-up period (76). Age should be one of the most important determinants of serum Vit D levels (15, 77).

However, another study showed no difference in age between cases with Vit D deficiency and those with normal Vit D levels (40). Moreover, a recently published study reported lower levels of Vit D in newly diagnosed children and adolescents with generalized epilepsy, with no difference between the two age groups (24). Therefore, considering the key role of Vit D in neuroprotection and brain development (49), we should pay more attention to the side effects of Vit D deficiency in patients with epilepsy.

It is estimated that more than 1 billion people in the world have Vit D deficiency (78). Mithal et al. (79) reviewed the status of Vit D in six regions of the world and reported that serum 25- (OH) D levels below 30 ng/mL are common in each area, and severe deficiency (< 25 nmoL/L) is most common in South Asia and the Middle East. Based on newly diagnosed epilepsy patients without ASM intervention, in terms of economic status and region, the prevalence of vitamin D deficiency in epileptic patients was highest in Europe (54.5, 95% CI: 27.4 -81.6%), followed by the Asian group (49.5, 95% CI: 35.6 -63.5%) and lowest in the American group (40.6, 95% CI: 13.9 -67.3%). Similarly, a recently published paper reviewed articles published in the past decade on the global status of Vit D, revealing that Vit D deficiency is a global health problem for all age groups (80). Girls and women from the Middle East have been shown to be particularly at risk (79, 80).

VitD3 is naturally converted from 7-dehydrocholesterol when exposed to sunlight in the epidermis (1). Despite sufficient sunlight, Vit D deficiency is still a common public health problem in many Asian countries (73, 81, 82); a considerable proportion of the population (about 22% of the urban and 90% of the rural) may be due to the lack of treatment facilities or lack of diagnostic knowledge, poverty, cultural beliefs and poor health service infrastructure (59). And there may be reasons such as geographical location and cultural practices based on food consumption affecting dietary Vit D supplementation (83), coupled with the lack of nutritional guidelines in Asia (e.g., the European Food Safety Authority) suggesting a lack of awareness and remedies (84). The lack of relevant diagnostic knowledge may lead to delayed diagnosis. Therefore, at the time of diagnosis, the patient may have had epilepsy for a long time, and changes in their lifestyle will affect bone health (85). The same is true for many other developing countries (86). These may lead to poor bone health before starting ASM treatment (85).

European and American countries (including Brazil and Portugal) are economically developed, with relatively complete infrastructure, such as public health and relatively developed education systems. Therefore, although they are located in the tropics, the prevalence of Vit D deficiency in patients with epilepsy in Europe and America is relatively lower than that in Asia. However, the difference was not statistically significant. The possible reason is that although Vit D supplementation has been implemented for many years in industrialized countries such as Brazil, and the overall prevalence of rickets has decreased, children from immigrant families are still at risk (87, 88). Since Brazil is a typical immigrant country, much its population is derived from the immigration wave in different historical periods. Immigrants and their descendants account for a considerable proportion of Brazil’s total population, so there are still many patients with Vit D deficiency. Moreover, it is reported that the prevalence of Vit D deficiency is high in low-latitude countries. Other studies have also shown that most newborns lack Vit D, even in tropical climates (89). These countries have more sufficient UV B radiation throughout the year, and as global warming continues to increase each year, humidity gradually increases. Therefore, sun-seeking behavior is not common (90), so many people choose to stay indoors with air conditioning (91), and may be associated with spending more time in front of the TV and computer indoors while using sunscreen with a high sun protection factor (SPF), air pollution, reducing sun exposure (92). In northern countries, the winter is more extended (92–94). Vit D deficiency is associated with dark skin, female, infants receiving less sunlight and exclusive breastfeeding, premature infants, patients with chronic diseases (skin diseases, malabsorption, cholestasis, renal insufficiency, disability), vegan diet, older age, and many other factors (use of chronic drugs including antiepileptic drugs and glucocorticoids) (95).

However, after ASM intervention, in terms of economic status and region, the prevalence of vitamin D deficiency in epileptic patients was highest in Europe (61, 95% CI: 49.3–72.8%), followed by the Asian group (46.3, 95% CI: 35.1–57.5%) and lowest in the American group (37.7, 95% CI: 20.9 -54.5%). High parental education is an independent predictor of good epilepsy knowledge (96), and high parental education can influence beliefs about the use of epilepsy drugs (97), according to a report. It is well known that epilepsy drugs reduce Vit D levels. Vit D deficiency is common in patients with epilepsy using ASM (11), because some ASMs interfere with the metabolism of Vit D (11) and other vitamins (98). The prevalence of Vit D deficiency is quite high among healthy subjects in industrialized countries (12). The reasons for the high prevalence of Vit D deficiency in the population include skin pigmentation, clothing, lower sunlight exposure time, genetic factors, and vegetarian factors.

Whether it is a single drug treatment or a multi-drug treatment, regardless of age, children with epilepsy may lack Vit D during the follow-up period related to the duration of ASM treatment, even if the Vit D level is normal at the beginning (99). Compared with monotherapy, polytherapy is associated with a high risk of bone mineral metabolism abnormalities (100). Consistent with our statistical results, the different treatment methods of ASM are divided into monotherapy and polytherapy. The prevalence of Vit D deficiency in epilepsy patients treated with a single drug (46.5%) is lower than that in epilepsy patients treated with multidrug (51.6%).

Polytherapy negatively affects Vit D status and bone mineral density (101). It has been reported that using an independent ASM type of comprehensive treatment has a negative impact on bone mineral density and Vit D (101, 102). Other reports have shown that although the type of ASMs does not affect Vit D status or bone mineral density, polytherapy has a negative effect on bone mineral density. Therefore, compared with monotherapy or shorter treatment, patients with comprehensive treatment or more prolonged treatment time (more than 2 years) of ASM will cause a more significant decrease in Vit D level (102). Similarly, Lee et al. (55) studied the longitudinal changes of Vit D levels in 143 pediatric patients receiving ASM treatment. Multidrug therapy and longer duration of treatment showed that the negative effect of 25-(OH)D changes was higher than that of monotherapy (*p* < 0.01) and shorter ASM duration (*p* < 0.01) (55). Therefore, polytherapy and long duration of ASM are independent risk factors for longitudinal reduction of 25- (OH) D (55). Moreover, a study conducted on Malaysian pediatric patients with epilepsy found that patients who received multiple treatments were more likely to lack Vit D than other patients (66). Nettokoven et al. (103) reported that polytherapy was associated with lower levels of Vit D compared to monotherapy. Previous studies have confirmed that multi-drug therapy is a risk factor for Vit D deficiency in patients with epilepsy (55, 103, 104).

However, there have also been experiments showing that patients receiving a single treatment have levels of Vit D similar to those receiving multiple therapies (105). At the same time, experiments by SERF et al. (42) showed that the use of three or more ASMs resulted in a 7.4 ng/mL increase in 25-(OH)D compared with patients using two or more ASMs. Obviously different from previous studies, a higher number of ASMs is associated with a higher level of 25-(OH)D (12). However, this result may be because our patients take more NEIASMs than EIASMs for multiple ASMs and are, therefore, less likely to be affected by EIASM-related VitD deficiency.

Studies have shown that, as vitamin deficiency is common in patients with epilepsy and may at least partially contribute to an increased fracture risk in this population, vitamin D monitoring should be considered as part of the routine management of patients with epilepsy (11). Several theories on the mechanism of ASM-associated bone disease have been proposed, but only some of the reported findings are explained. The most common explanation is that ASMs such as carbamazepine that induce hepatic cytochrome P450 enzymes cause increased conversion of Vit D to inactive metabolites in the liver microsomes. The decreased biologically active Vit D leads to decreased calcium absorption in the gut, resulting in hypocalcemia and increased circulating PTH (42). Hyperparathyroidism leads to increased bone resorption, reduced bone mineral density (BMD), and increased fracture risk (106, 107).

## 5 Article advantages

The advantages of this study include several key points. First of all, we want to understand whether epilepsy itself is related to Vit D status, so we mainly study new epilepsy patients who have not yet begun any drug treatment. This article represents the first and most comprehensive systematic review and meta-analysis of the prevalence of Vit D deficiency in patients with epilepsy. This study included more participants who did not have baseline Vit D measurements before starting any ASM. Thus, a more reliable prevalence estimate was obtained, and confidence in the study results increased. Secondly, subgroup analysis was performed considering diagnostic criteria, age, economic development level and region, single drug treatment, and multidrug treatment comparison. This method helps to reduce the heterogeneity between studies. It provides a more scientific and reasonable method to evaluate the results of the prevalence of Vit D deficiency in different subgroups. In addition, the potential sources of heterogeneity and influencing factors of the incidence of VitD deficiency. This will help better understand the changes observed in the prevalence estimates and gain insight into the relevant factors leading to the occurrence of Vit D deficiency in patients with epilepsy. Overall, the advantages of this study lie in its comprehensiveness, careful classification and analysis of factors, and identification of potential sources of heterogeneity. These aspects contribute to a more comprehensive understanding of the prevalence of Vit D deficiency in patients with epilepsy.

## 6 Limitations

There are several limiting factors to be considered in this study. First, we did not summarize the effects of biochemical markers of bone turnover. We should also consider calcium, phosphorus, bone, alkaline phosphatase, parathyroid hormone, osteocalcin, and bone mineral density to determine the antiepileptic drugs’ impact indirectly. Studies have shown that if patients do not undergo bone health screening, they will miss most patients with low 25-(OH)D levels and will continue to use ASM, which will prove to be harmful (20). Any changes in bone mineral density (BMD) measurements were observed only after 18 months of treatment. Therefore, the evaluation of biochemical and hormonal parameters is critical. However, it should be noted that the measurement of total alkaline phosphatase may only reflect liver metabolism, so it is not an appropriate marker of bone metabolism (108). So, we should have taken it into account. Secondly, we did not evaluate the effect of antiepileptic drug types (enzyme-induced and non-enzyme-induced antiepileptic drugs) on Vit D levels, nor did we count the effect of VitD supplementation on Vit D levels in patients with epilepsy. Some literature supports the impact of specific drugs and Vit D supplementation on Vit D content in patients. Still, there is no specific statistical Vit D content, and other indirect ways are used to prove the effect of drugs on VitD content in patients with epilepsy, so they are not adopted. Moreover, we did not assess the effect of seasonal variations on measured Vit D concentrations. We also did not evaluate the dietary intake of Vit D and its impact on Vit D concentration in patients. In addition, due to several limitations, a single comprehensive estimate of prevalence should be interpreted cautiously. First, there is heterogeneity even in subgroup analysis, which is often difficult to avoid in epidemiological studies. Secondly, the difference in prevalence between studies may be due to factors such as sample source, different definitions of Vit D deficiency, sample representativeness, and specificity of diagnostic tools. Many studies rely on clinical history and symptoms to diagnose epilepsy, which may lead to underdiagnosis and overdiagnosis. Third, some literature data are not comprehensive enough, such as Table 1 fails to reflect the diagnostic criteria of epilepsy, the method of measuring Vit D, etc. Some literature was excluded from the subgroup analysis due to the lack of specific values of Vit D content, and the amount of literature included in the subgroup analysis may have some bias in the results. In addition, it is essential to note that this study excludes unpublished articles, which may lead to underestimation or overestimation of the true prevalence of Vit D deficiency in epilepsy. Finally, differences in individual characteristics in the study may affect the results. Although this study provides valuable insights, these limitations should be considered when interpreting the findings.

## 7 Conclusion

In conclusion, our results show that nearly half of patients with epilepsy have Vit D deficiency, with a prevalence of 50.2%, which strengthens the high proportion of Vit D deficiency in patients with epilepsy. Our analysis showed that diagnostic criteria, age, level of economic development, and region may not be associated with epilepsy comorbid Vit D deficiency. The high prevalence of insufficient Vit D levels in children with epilepsy is significant because these children have an additional risk of bone damage due to comorbid neuromotor dysfunction and long-term treatment (24).Given the high prevalence of Vit D deficiency in epilepsy, it is recommended that patients with these related factors be followed up regularly for a long time. In addition, screening Vit D levels is simple, inexpensive, and has no significant side effects. Therefore, it is emphasized that monitoring Vit D levels is part of the routine management of patients with epilepsy and may improve the bone health of this vulnerable population. Finally, to clarify the role of Vit D in children with epilepsy, large-sample multicenter, long-term trials and basic research are needed in the future, emphasizing the potential importance of the potential etiology of epilepsy as a risk factor for Vit D deficiency. Further studies are needed to evaluate the effects of Vit D supplements.

## Data Availability

The original contributions presented in the study are included in the article/Supplementary material, further inquiries can be directed to the corresponding author/s.
